# Volatile Fingerprint of Italian Populations of Orchids Using Solid Phase Microextraction and Gas Chromatography Coupled with Mass Spectrometry

**DOI:** 10.3390/molecules19067913

**Published:** 2014-06-11

**Authors:** Alessandra Manzo, Sara Panseri, Ilda Vagge, Annamaria Giorgi

**Affiliations:** 1 Centre for Applied Studies in the Sustainable Management and Protection of the Mountain Environment—Ge.S.Di.Mont.-Università degli Studi di Milano, Via Morino 8, 25048 Edolo, Brescia, Italy; E-Mails: alessandra.manzo@unimi.it (A.M.); anna.giorgi@unimi.it (A.G.); 2 Department of Veterinary Science and Public Health- Università degli Studi di Milano, Via Celoria 2, 20133 Milan, Italy; 3 Department of Agricultural and Environmental Sciences - Production, Landscape, Agroenergy—Università degli Studi di Milano, Via Celoria 2, 20133 Milan, Italy; E-Mail: ilda.vagge@unimi.it

**Keywords:** *Orchidaceae*, Italy, conservation, floral scent, HS/SPME GCMS

## Abstract

The volatile fraction of *Ophrys sphegodes* Mill. subsp. *sphegodes*, *Ophrys bertolonii* subsp. *benacensis* (Reisigl) O. Danesch, E. Danasch & Ehrend. and *Neotinea tridentata* (Scop.) R.M. Bateman, Pridgeon & M.W. Case, three orchid species with different pollinator attraction strategies, sampled *in vivo* and *in situ*, were evaluated by headspace solid phase microextraction coupled with gas-chromatography and mass spectrometry. The results were compared with the volatile compounds emitted by flowering plant samples picked from the same populations of orchid species. Hydrocarbons, aldehydes, alcohols and terpenes were the major constituents of “*in vivo*” orchid scents and some distinctive differences in volatile metabolite composition were observed between *Ophrys* and *Neotinea* species. Moreover, the odour bouquets of the picked flowering plant samples were different from the *in vivo* ones and in particular different proportions of the various terpenes and an increase of α-pinene were observed. In conclusion HS/SPME GCMS proved to be a suitable technique for analyzing and distinguishing the volatile fingerprint of different orchid species, sampled *in vivo* and *in situ* in a non-disruptive way, with potentially great advantages for ecophysiological studies of rare and endangered species.

## 1. Introduction

Investigation into plant and flower scents represents an important field of modern research directed at special biological recognition theories. The scent of a flower is often a complex blend of secondary volatile metabolites, and together with colour and shape is considered to be the main signal attracting pollinators [1,2,3,4], which in turn affect the reproductive performance of plants, their relationships with the environment and, therefore, their conservation (especially for rare and endangered species). 

In particular, the variety of shapes, colours and scents present in orchids is seldom found in other plant families. These characteristics contribute to the unique strategies used by orchids to attract pollinators. Many orchid species provide nectar, but other species are deceptive, so they attract pollinators in different ways, the most common strategies being mimicry of nectariferous flowers (e.g., *Orchis*, *Neotinea*, *Anacamptis*), sexual deception (e.g., *Ophrys*) and provision of shelter (e.g., *Serapias*) [5]. 

In deceptive species attractiveness is very important to ensure reproductive success. For instance, orchid species of the genera *Ophrys* and *Neotinea* are known to produce complex bouquets of volatiles typically consisting of more than 100 chemical compounds [6,7,8]. The species belonging to these genera are all deceptive, but *Ophrys* species use a sexual deception strategy, while *Neotinea* is a food-deceptive genus [5]. The *Ophrys* species attract and deceive their pollinators through an elaborate sexual mimicry that involves visual cues and volatile semiochemicals that mimic the pheromone of female insects. Food-deceptive orchids mimic rewarding species, some with specific models (e.g., *Disa*) others with flowers that have the typical characteristics of rewarding plants (*i.e.*, nectar guides, spur, *etc.*) (e.g., *Orchis*, *Neotinea*, *Anacamptis*) [9]. Moreover, *Ophrys* species have evolved a high degree of pollinator specificity, while *Neotinea* species are more generalist.

Despite the importance of volatile compounds for the ecophysiological aspects of plant life, in the literature there are only a few publications on the characterization of orchids’ volatile profiles [10,11]. Flower, as well as whole plant, scents are conventionally analyzed by different methods, usually based on solvent extraction, steam distillation, or supercritical fluid extraction, that use destructive approaches. Moreover, the methods so far applied require that flowers or plants are collected, causing stress and mechanical damage to the plants thus altering their volatile profiles. Following these considerations soft extraction methods are preferred and HS-SPME was chosen to analyze the orchid samples. This technique has been developed for the fractionation of volatile organic compounds in several matrices, but can be also applied for the direct sampling of flower scents [10,12,13]. The method is rapid, solvent-free and inexpensive and reduces sampling stress; moreover it may be easily transferred into the field in order to investigate real *in vivo* volatile emission.

The aim of this study was to characterize, for the first time, the volatile organic compounds emitted, *in vivo*, by *Ophrys sphegodes* Mill. subsp. *sphegodes*, *Ophrys bertolonii* subsp. *benacensis* (Reisigl) P. Delforge and *Neotinea tridentata* (Scop.) R.M. Bateman, Pridgeon & M.W. Case, three Italian populations of orchid species with different attraction strategies, sampling them *in situ*, in a non-disruptive way. Moreover, in order to define the most reliable sampling approach for improving knowledge regarding orchids’ ecophysiological aspects, a comparison was made with the volatile profile obtained from flowering plant samples picked from the same orchid populations. 

## 2. Results and Discussion

### 2.1. Analysis of “in Vivo” VOCs

The volatile compounds emitted by *O. sphegodes* subsp. *sphegodes*, *O. bertolonii* subsp. *benacensis* and *N.* *tridentata* species are listed in Table 1. We identified 67, 93 and 77 compounds, respectively. These VOCs belonged to the major chemical classes, such as hydrocarbons, aldehydes, ketones, alcohols, furans, phenols, free fatty acids and terpenes. As reported in Table 1, the aromatic profiles of the three orchid species showed some distinctive differences.

The difference among VOCs extracted and detected from picked samples respect to “*in situ*” arisen from two variables. Firstly by sampling “*in situ*” we observe a general dilution factor of monitored headspace respect the vials (20 mL) because the special equipment is specially designed to promote air circulation to the contained plant during the SPME extraction. The second reason can take place to the cut operations made in order to collect the flowers. Some terpenes are contained into special plant organ, that damaged, are released in a headspaces vial.

**Figure 1:**
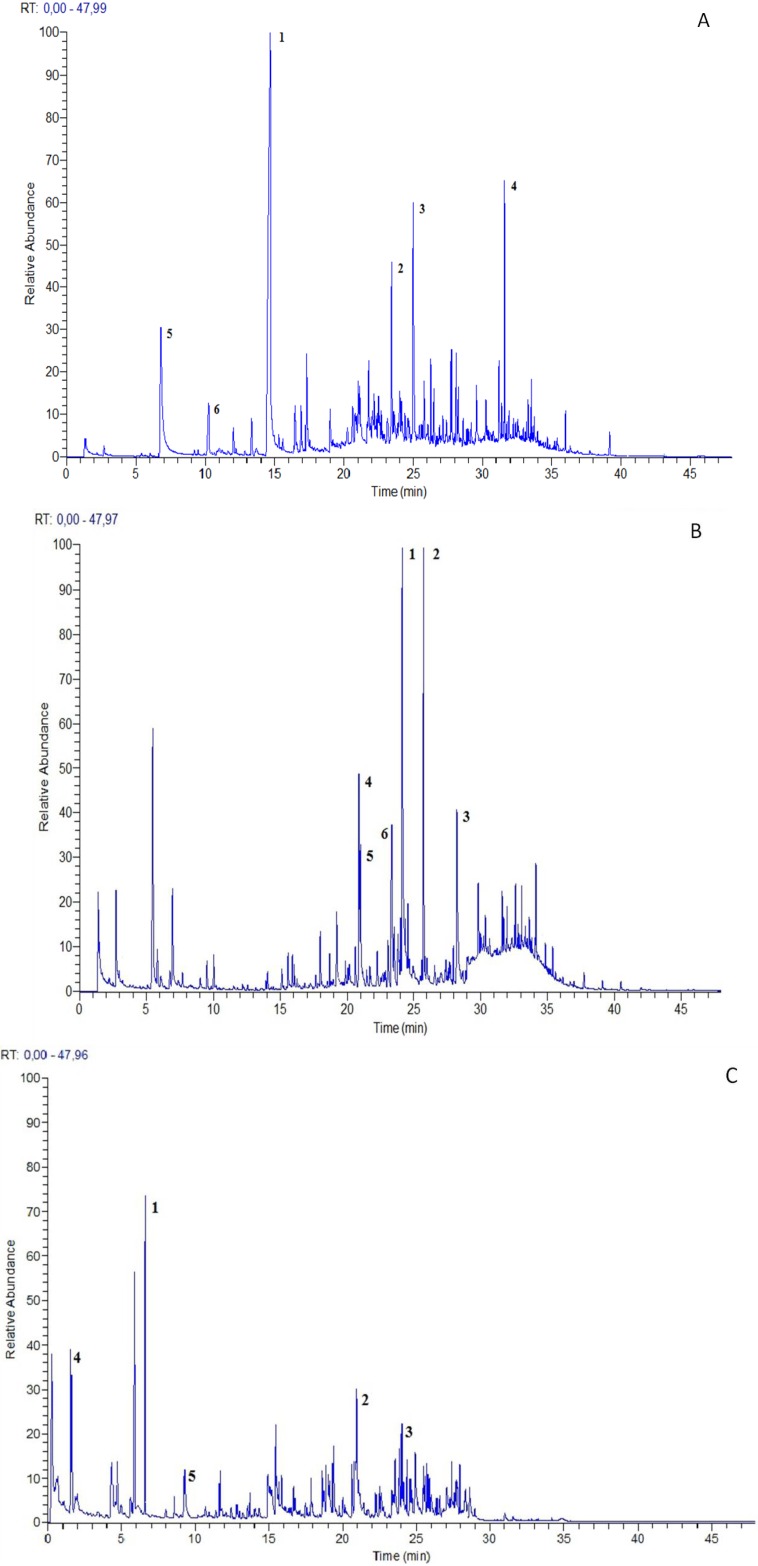
Volatile fingerprint of *O. sphegodes* subsp. *sphegodes* (**A**), *O. bertolonii* subsp. *benacensis* (**B**) and *N*. *tridentata* (**C**) “*in vivo*” plant (the most representative compound peaks were labeled with numbers).

The main components of *O. sphegodes* subsp. *sphegodes* bouquet were represented by terpenes, free fatty acids and phenols, followed by alcohols, aldehydes and hydrocarbons in minor amounts. In particular, as reported in Table 1 and Figure 1, the most representative compounds detected were d-limonene (22.13%), formic acid (11.70%), α-zingibirene (9.98%), phenol (9.65%), α−pinene (4.65%) and undecane (3.56%). Among terpenes, in minor amounts, the presence of β−pinene (2.19%) and cyclosativene (2.47%) was revealed. Terpenes, ketones, and aldehydes, were the major constituents of *O. bertolonii* subsp. *benacensis* aroma, followed by hydrocarbons and alcohols. In particular 3,5-octadien-2-one (9.85%), caryophillene (6.73%), dodecanal (4.90%), 4-methyltetradecane (4.69%), decanal (3.48%) and nonanal (3%) were the main compounds (Table 1 and Figure 1). 

*O. sphegodes* subsp. *sphegodes* and *O. bertolonii* subsp. *benacensis* are sexually deceptive orchids and numerous behavioral tests have shown that the *Ophrys*-pollinator relationship is highly specific: each *Ophrys* species is pollinated by males of usually only one or a few pollinator species [14,15,16,17]. In particular the pollinators of *O. sphegodes* subsp. *sphegodes* are small bees of the genus *Andrena*, especially *A. nigroaena* and *A. limata*, while *O. bertolonii* subsp. *benacensis* *O. bertolonii* subsp. *benacensis* is characterized by higher pollinator specificity with the main pollinator being the hymenopterous *Chalicodoma parietina* [18]. 

**Table 1:** Identification of volatile organic compounds by HS-SPME-GCMS from “*in vivo*” orchids.

		Control		*O. sphegodes* subsp. *sphegodes*		*O. bertolonii* subsp. *benacensis*			*N. tridentata*				
		
Compounds	RT ^a^	Mean ( *n* = 3) ^b^	SD ^c^	Mean ( *n* = 3) ^d^	SD ^c^	Mean ( *n* = 3) ^d^	SD ^c^		Mean ( *n* = 3) ^d^		SD ^c^		
hexane	1.50	0.96	0.01	ND	-	ND	-		7.14^4^		0.03		
octane	2.21	0.39	0.14	0.16	0.13	0.08	0.08		0.14		0.00		
butane	2.26	ND	-	ND	-	0.17	0.17		1.09		0.15		
1-octene	2.53	ND	-	ND	-	0.17	0.17		0.22		0.00		
nonane	3.25	ND	-	ND	-	0.22	0.22		1.36		0.32		
undecane	10.25	0.73	0.65	3.56^6^	0.00	ND	-		0.76		0.01		
1-tridecene	11.85	0.07	0.00	ND	-	0.11	0.11		ND		-		
dodecane	14.88	0.13	0.02	ND	-	1.39	1.00		ND		-		
tridecane	18.72	ND	-	0.11	0.00	ND	-		0.79		0.03		
4-methyl-tetradecane	20.55	ND	-	ND	-	4.69^4^	0.53		1.25		0.11		
pentadecane	21.43	0.68	1.68	ND	-	1.20	0.57		0.86		0.15		
nonadecane	22.47	0.73	0.00	0.71	0.00	0.21	0.19		ND		-		
heptadecane	25.98	0.25	0.01	ND	-	0.73	0.57		ND		-		
2-tridecane	29.86	0.59	0.00	ND	-	0.43	0.26		0.50		0.00		
**Total**		**4**.**53**		**4**.**54**		**9**.**4**			**14**.**11**				
*Aldehydes*													
pentanal	4.78	ND	-	ND	-	0.15	0.16		0.11		0.01		
esanal	9.02	1.48	1.97	0.42	0.34	0.64	0.44		0.33		0.03		
3-hexenal	12.22	0.12	0.01	ND	-	0.50	0.36		0.16		0.10		
heptanal	13.98	0.10	0.01	ND	-	0.65	0.81		0.15		0.01		
3-methyl-2-butenal	14.51	0.48	0.27	ND	-	0.28	0.08		ND		-		
2-hexenal	15.27	1.25	0.00	0.42	0.02	0.67	0.44		1.39		0.89		
octanal	18.01	ND	-	ND	-	1.83	0.49		0.29		0.00		
2-heptanal	18.89	ND	-	ND	-	0.34	0.31		ND		-		
nonanal	21.02	0.72	1.60	1.68	0.79	3.00^6^	0.93		7.92^2^		1.11		
2-octenal	21.74	ND	-	ND	-	0.16	1.90		ND		-		
decanal	23.63	0.72	0.64	0.86	0.02	3.48^5^	0.07		7.42^3^		0.78		
2-nonanal	24.13	ND	-	1.08	0.72	0.58	0.41		ND		-		
undecanal	25.86	0.05	0.30	ND	-	0.29	0.67		0.64		0.03		
benzene acetaldehyde	26.43	ND	-	ND	-	0.05	0.04		0.06		0.01		
dodecanal	28.00	0.40	1.23	0.46	0.30	4.90^3^	0.79		ND		-		
2-ethylbenzaldehyde	28.33	0.26	0.21	ND	-	0.11	0.00		ND		-		
**Total**		**5.59**		**4.92**		**17.63**			**18.47**				
*Esters*													
ethyl acetate	3.01	0.96	0.04	0.09	0.00	ND	-		0.69		0.01		
2-methylbutanoic acid methyl ester	5.85	ND	-	ND	-	ND	-		12.36^2^		0.21		
3-hexen-1-ol-acetate	19.00	0.48	0.12	1.64	0.24	1.69	0.21		0.34		0.02		
geranyl acetate	30.64	0.33	0.00	ND	-	0.11	0.07		0.55		0.06		
**Total**		**1.77**		**1.73**		**1.8**			**13.93**				
*Ketones*													
3-penten-2-one	5.11	ND	-	ND	-	0.54	0.54		2.04		1.12		
2-nonen-4-one	15.93	1.17	0.10	ND	-	2.32	1.88		1.84		0.67		
2-methyl-6-heptanone	16.21	0.70	0.00	ND	-	0.12	0.08		ND		-		
3-octanone	16.78	ND	-	0.18	0.18	0.26	0.15		0.12		0.00		
acetoin	17.6	2.42	1.58	ND	-	0.52	0.67		ND		-		
2-octanone	17.86	0.32	0.09	0.22	0.24	0.81	0.49		0.20		0.03		
1-octen-3-one	18.39	0.37	0.00	ND	-	0.64	0.47		ND		-		
pentadecanone	23.83	ND	-	0.33	0.00	1.26	1.78		0.06		0.00		
3.5-octadien-2-one	23.92	ND	-	0.3	0.12	9.85^1^	1.50		0.71		0.11		
pantoic lactone	24.39	ND	-	ND	-	0.56	0.31		ND		-		
2-pentadecanone	32.8	ND	-	ND	-	0.44	0.62		0.25		0.15		
**Total**		**4.97**		**1.03**		**17.3**			**5.22**				
*Phenols*													
anisole	19.46	0.11	0.05	ND	-	0.39	0.36		0.16		0.01		
4-methylanisole	22.04	ND	-	1.01	0.60	0.09	0.07		ND		-		
phenol	31.61	ND	-	9.65^4^	0.44	0.39	0.41		ND		-		
4-methylphenol	33.29	0.07	0.00	0.77	0.04	3.53	2.77		ND		-		
**Total**		**0.18**		**11.43**		**4.4**			**0.16**				
*Alcohols*													
ethanol	3.81	0.11	0.00	ND	-	0.11	0.16		ND		-		
1-nonanol	4.03	0.21	0.00	ND	-	0.26	0.28		1.16		0.19		
2-methyl-3-buten-2-ol	7.42	1.32	0.05	ND	-	ND	0.28		1.46		0.89		
2-ethyl-1-hexanol	8.43	0.36	0.10	ND	-	ND	-		0.09		0.00		
isobutanol	10.36	ND	-	ND	-	0.09	0.05		ND		-		
3-pentanol	11.17	ND	-	0.48	0.00	0.06	0.03		ND		-		
1-penten-1-ol	12.30	ND	-	0.02	0.00	ND	-		0.27		0.03		
1-butanol	12.75	0.07	0.00	ND	-	ND	-		0.09		0.00		
1-penten-3-olo	13.66	0.32	0.00	ND	-	0.36	0.31		0.36		0.11		
isoamylalcohol	15.47	0.10	0.01	0.04	0.00	ND	-		ND		-		
1-pentanol	17.00	ND	-	ND	-	0.52	0.65		0.03		0.00		
heptanol	19.20	0.22	0.17	1.64	0.28	ND	-		0.14		0.02		
3-hexen-1-ol	20.82	0.35	0.00	ND	-	2.19	0.44		ND		-		
2-nonanol	20.89	0.72	0.60	0.90	0.87	2.19	1.93		ND		-		
1-octen-3-ol	22.55	2.94	0.89	ND	-	0.57	0.26		0.59		0.56		
2-decanol	26.08	ND	-	0.38	0.00	ND	-		ND		-		
1-nonanol	27.04	ND	-	ND	-	0.13	0.18		1.29		0.77		
2-undecanol	28.27	0.26	0.21	0.61	0.48	0.03	0.33		0.48		0.21		
3-decen-1-ol	29.59	ND	-	1.49	0.00	ND	-		ND		-		
1-heptadecanol	30.46	ND	-	ND	-	0.42	0.23		0.31		0.08		
1-dodecanol	32.23	ND	-	0.49	0.04	0.14	0.13		ND		-		
isothymol	34.70	ND	-	0.19	0.00	ND	-		ND		-		
1.4-benzenediol	34.86	1.08	0.77	0.11	0.00	0.69	0.18		ND		-		
**Total**		**8.05**		**6.35**		**7.76**			**6.27**				
*Furans*													
2-methylfuran	3.19	ND	-	0.09	0.06	ND	-		ND		-		
4-ethylfuran	4.21	0.21	0.00	ND	-	0.04	0.06		0.24		0.03		
2-butylfuran	11.62	0.21	0.00	ND	-	ND	-		0.13		0.01		
**Total**		**0.42**		**0.09**		**0.04**			**0.37**				
*Terpenes*													
α-pinene	6.03	ND	-	4.65^5^	0.72	1.05	0.00		0.34		0.11		
thujene	6.38	0.23	1.01	0.35	0.00	ND	-		0.44		0.03		
β-pinene	9.61	0.08	0.00	2.19	0.00	0.17	0.25		4.59^5^		1.01		
sabinene	10.55	ND	-	1.31	0.00	ND	-		ND		-		
δ.3.carene	12.00	0.22	0.00	0.81	0.51	0.09	0.07		0.28		0.01		
β-myrcene	13.33	ND	-	1.22	0.30	ND	-		ND		-		
d-limonene	14.69	0.04	0.27	22.13^1^	1.68	0.15	0.31		ND		-		
sabinene	14.74	0.04	0.02	ND	-	ND	-		0.09		0.00		
terpene	16.33	0.92	0.00	ND	-	ND	-		0.47		0.03		
γ-terpinene	16.45	ND	-	1.09	0.97	ND	-		0.31		0.90		
*o*-cymene	16.91	ND	-	1.41	0.24	0.05	0.01		0.10		0.09		
*p*-cymene	17.36	0.33	0.06	0.23	0.18	0.20	0.29		0.32		0.07		
α−terpinolene	17.71	0.76	1.58	0.12	0.14	ND	-		0.57		0.08		
α-cyclocitral	18.56	ND	-	0.62	0.00	1.82	2.50		ND		-		
sequiterpene	21.65	ND	-	0.42	0.16	ND	-		ND		-		
isopatchoulane	22.66	ND	-	0.98	0.12	0.19	0.00		1.03		0.66		
*trans*-linalool oxide	22.88	0.05	0.00	0.34	0.00	0.94	0.66		0.06		0.18		
cyclosativene	23.11	0.30	0.00	2.47	1.75	0.28	0.21		ND		-		
copaene	23.40	ND	-	ND	-	1.11	0.46		0.81		0.00		
isolongifolene	23.88	ND	-	1.46	0.12	ND	-		ND		-		
4-thujanol	24.57	ND	-	ND	-	1.09	1.14		ND		-		
linalool	24.71	ND	-	0.59	0.60	0.16	0.23		ND		-		
α-zingibirene	24.93	0.07	0.01	9.98^3^	7.15	0.26	0.11		0.26		0.90		
sequiterpene	25.09	ND	-	0.90	0.31	ND	-		0.46		0.12		
α-bergamottene	25.49	0.22	0.00	ND	-	0.55	0.00		ND		-		
caryophillene	25.60	ND	-	ND	-	6.73^3^	1.84		0.69		0.47		
4-terpineol	25.79	1.19	0.41	2.06	0.43	1.28	1.18		0.65		0.01		
menthol	26.53	0.20	2.54	2.02	1.06	0.90	1.14		0.14		0.03		
verbenone	27.66	0.24	0.00	0.55	0.45	0.60	0.47		0.25		0.01		
eucarvone	27.77	0.20	0.01	1.44	1.27	0.19	0.19		ND		-		
naphtalene	28.91	0.14	0.00	1.12	1.04	0.20	0.09		0.50		0.04		
sesquiphellandrene	29.09	ND	-	ND	-	2.44	0.38		ND		-		
estragol	30.07	0.28	0.35	ND	-	0.37	0.89		0.35		0.11		
piperitone oxide	31.39	ND	-	0.63	0.00	0.77	0.41		ND		-		
ascaridole	31.78	ND	-	0.37	0.07	ND	-		ND		-		
α-santolene	33.74	ND	-	0.47	0.00	ND	-		ND		-		
**Total**		**5.51**		**61.92**		**21.58**			**12.72**				
*Free fatty acids*													
acetic acid	22.26	0.62	0.00	0.31	0.35	0.71	0.00		1.33		0.02		
formic acid	23.44	0.69	0.08	11.7^2^	0.18	ND	-		ND		-		
pivalic acid	25.21	ND	-	ND	-	0.3	0.09		ND		-		
hexanoic acid	30.34	ND	-	0.32	0.21	0.42	0.00		0.15		0.00		
heptanoic acid	31.99	0.21	1.17	ND	-	0.45	0.29		0.35		0.11		
octanoic acid	33.08	ND	-	0.03	0.00	0.19	1.01		0.67		0.03		
nonanoic acid	34.14	0.14	0.49	0.39	0.00	1.33	0.74		0.25		0.12		
decanoic acid	35.41	ND	-	ND	-	0.84	0.00		0.91		0.00		
benzoic acid	37.74	0.12	0.41	0.49	0.49	0.42	0.13		0.40		0.02		
**Total**		**1.77**		**13.25**		**4.66**			**4.05**				
*Miscellaneous*													
acetonitrile	5.42	8.04	0.32	0.42	0.36	11.95	0.12		17.26		0.98		
dimethyl sulfone	31.18	0.45	0.00	1.24	0.18	0.30	0.21		0.06		0.00		
**Total**		**8.49**		**1.65**		**12.25**			**17.32**				

^a^ Retention time; ^b^ Normalized amount of volatile compounds (percentage) (peak of volatile compound/total peak area of all volatile compounds) of control samples (*n* = 3); ^c^ Standard deviation (±); ^d^ Normalized amount of volatile compounds (percentage) (peak of volatile compound/total peak area of all volatile compounds) of *O. sphegodes* subsp. *sphegodes*, *O. bertolonii* subsp. *benacensis* and *N. tridentata* living plants (*n* = 3); ND: not detected; The most representative compounds for all orchid species were labeled with a number.

*Ophrys* flowers usually produce complex bouquets of volatiles, but presumably not all components are important for the attraction of male pollinators [6]. The literature reports that only some hydrocarbons, in particular very long-chain alkanes and alkenes and terpenes act as chemical mimicry of the sex pheromone of the virgin female pollinators. In agreement with our results, other authors confirmed terpenes and hydrocarbons together with alcohols and aldehydes as major constituents of orchid volatile profiles [10,11,19,20] and among these chemical classes a high emission of nonanal and caryophyllene by orchid flowers was previously reported [19,21,22]. In particular, the chemical composition of orchids pollinated by bees (e.g., *Ophrys*) revealed an abundance of terpenes (mono- and sesquiterpenes) [23]. 

Not all hydrocarbons, aldehydes and alcohols make an important contribution to the aroma of the plants. Some of them are abundant in cuticular waxes and are said to have the primary function of protecting the plant from dehydration. Other compounds seem to play an important role in plant-herbivore interaction and acting as a solvent for the male-attracting volatiles [24]. 

The aromatic profile of *N. tridentata* was characterized by a high concentration of aldehydes, hydrocarbons, esters and terpenes and in particular, as reported in Table 1 and Figure 1, 2-methylbutanoic acid methyl ester (12.36%), nonanal (7.92%), decanal (7.42%), hexane (7.14%) and β-pinene (4.59%). 

The genus *Neotinea* includes food-deceptive species and, in contrast with the *Ophrys* genus, is thought to attract and deceive mostly naive pollinators by generally mimicking nectariferous plants [5]. *N. tridentata* is quite generalist in terms of pollinators which are different species of hymenoptera, in particular solitary bees (e.g., *Osmia bicolor*), *Apis mellifera* and coleoptera (e.g., *Cleridae*) [25]. In this genus colour is generally regarded as a primary cue to attract insects to food-deceptive flowers but in this study several odour compounds were found in the floral scents of *N. tridentata* [5]. As reported in Table 1 most of the VOCs were common to *O. sphegodes* subsp. *sphegodes* and *O. bertolonii* subsp. *benacensis* flowers but different proportions of the various compounds were observed between *Oprhys* and *Neotinea* species. In particular, a minor content of terpenes was identified in *N. tridentata* bouquet and this difference can be related to the different pollinator attraction strategies. However, there is no confirmation that floral odour is not of importance in pollinator attraction in food-deceptive species, indeed it was observed that in *Anacamptis morio*, a food-deceptive species*,* scent emission elicit a response in bee antennae [26].

### 2.2. Analysis of Picked Flowering Plant VOCs

**Figure 2:**
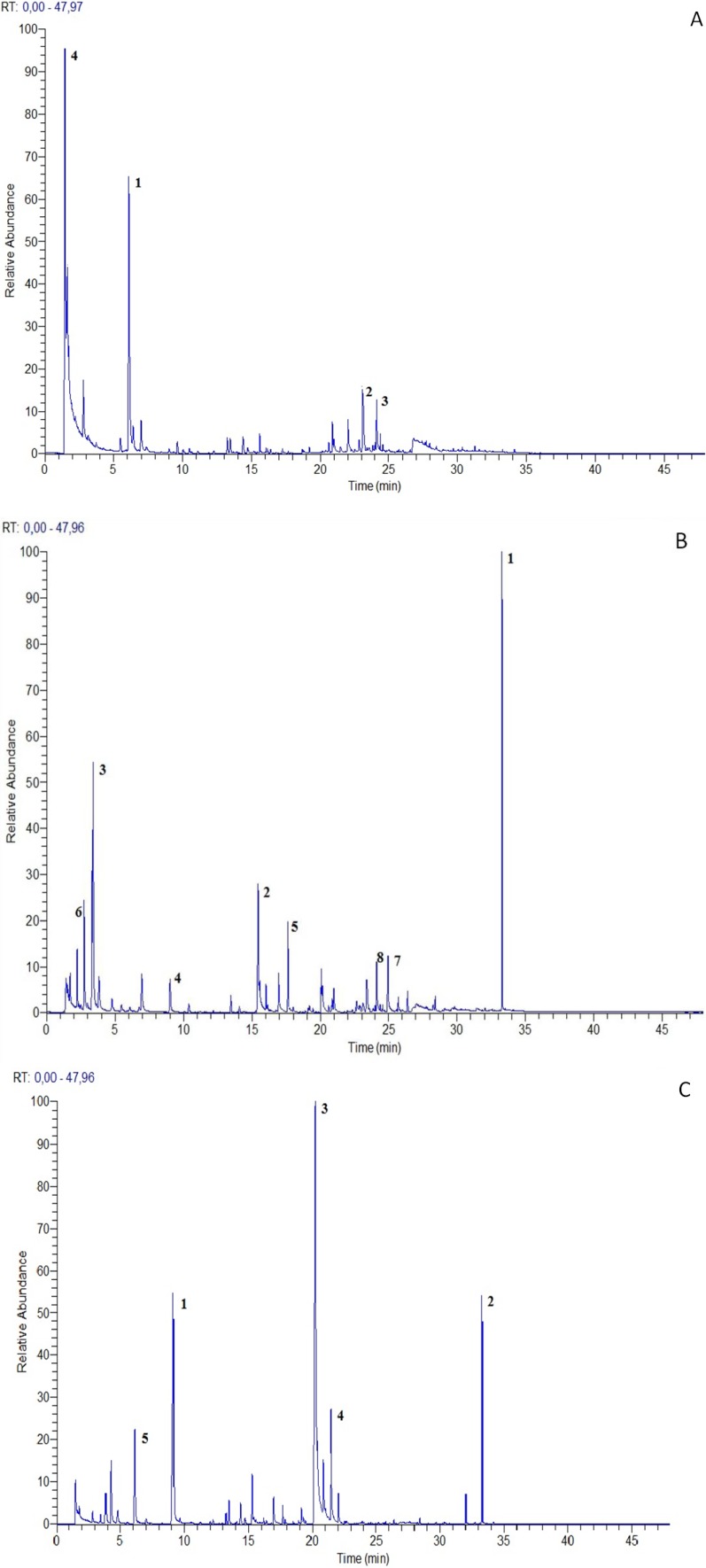
Volatile fingerprint of *O. sphegodes* subsp. *sphegodes* (**A**), *O.* *bertolonii* subsp. *benacensis* (**B**) and *N. tridentata* (**C**) picked flowering plants (the most representative compound peaks were labeled with numbers).

The volatile profile of *O. sphegodes* subsp. *sphegodes*, *O. bertolonii* subsp*. benacensis* and *N. tridentata* picked flowering plant samples consists of 56, 53 and 63 compounds, respectively (Table 2). As observed in “*in vivo*” orchids the aromatic profiles of the three picked flowering plant samples showed some distinctive differences in volatile fingerprint and the most representative compounds of all species were reported in Table 2 and Figure 2. 

Comparing the VOC profiles of “*in vivo*” and picked flowering plant samples of the same orchid species we showed some differences in secondary metabolite composition. In particular we observed an increased content of phenols and alcohols in *O. bertolonii* subsp. *benacensis* and *N. tridentata* picked flowering orchid samples compared to living plants. Among these chemical classes we observed a large amount of 4-methylphenol in both orchid species (13,25% and 11,28% respectively) and of isoamylalcohol (13.03%) and 3-hexen-1-ol (8,52%) respectively in *O. bertolonii* subsp. *benacensis* and *N. tridentata* fingerprint*.*

An increase in hydrocarbons was also revealed in *O. sphegodes* subsp. *sphegodes* and *O. bertolonii* subsp. *benacensis* flowering plant samples and hexane, butane and nonane were considered the most representative compounds. Finally, we observed that flowering plant aromas showed a high content of terpenes. In particular in this case the most representative compounds were α-pinene (47.95%), thujene (3.05%) and cyclosativene (6.38%), followed in lesser amounts by β-myrcene (2.07%). 

Collecting plant tissues and flowers definitely impacts on the volatile profiles, causing dramatically alterations if compared with *in vivo* and *in situ* VOC samples. These alterations can be related to the mechanical damage provoked by the collection of plants [27] and in particular in this study different proportions of the various terpenes were observed and a large increase of α-pinene was detected especially in *O. sphegodes* subsp. *sphegodes* fingerprint. These data were confirmed also by literature [27].

**Table 2:** Identification of volatile organic compounds by HS-SPME-GCMS from picked flowering orchid samples.

								*O. sphegodes* subsp. *sphegodes*							*O. bertolonii* subsp. *benacensis*									*N. tridentata*											
			
		Compounds			RT ^a^			Mean (*n* = 3) ^b^				SD ^d^			Mean (*n* = 3)					SD				Mean (*n* = 3)				SD							
		*Hydrocarbons*																																	
		hexane			1.50			3.87^4^				1.00			0.41					0.10				0.20				0.00							
		2-methylbutadiene			1.64			2.30				0.89			0.23					0.00				ND				-							
		butane			2.26			ND				-			6.93^6^					3.11				0.10				0.05							
		nonane			3.25			ND				-			10.87^3^					1.31				0.06				0.01							
		1-tridecene			11.85			ND				-			ND					-				0.24				0.00							
		tridecane			18.71			0.33				0.02			ND					-				0.15				0.00							
		dodecane			18.79			0.17				0.01			ND					-				ND				-							
		pentadecane			21.45			0.26				0.03			ND					-				7.52^4^				1.60							
		nonadecane			22.47			0.23				0.00			0.33					0.07				0.37				0.34							
		heptadecane			25.98			0.13				0.02			0.17					0.00				0.06				0.00							
		**Total**						**7.56**							**18.95**									**11.09**											
		*Aldehydes*																																	
		pentanal			4.78			ND				-			1.30					0.24				1.87				1.01							
		esanal			9.02			0.48				0.04			8.36^4^					1.80				17.95^1^				2.75							
		3-hexenal			12.22			0.28				0.02			ND					-				0.31				0.21							
		heptanal			13.94			0.12				0.01			0.06					0.02				0.89				0.00							
		2-hexenal			15.27			0.04				0.00			0.51					0.00				2.43				0.67							
		octanal			18.01			0.08				0.01			0.32					0.01				ND				-							
		2-heptanal			18.87			ND				-			0.15					0.05				0.25				0.01							
		nonanal			21.00			0.40				0.03			2.31					0.69				ND				-							
		2-octenal			21.74			ND				-			ND					-				ND				-							
		decanal			23.60			ND				-			0.15					0.03				ND				-							
		2-nonanal			24.13			5.39^3^				0.98			2.88					1.59				ND				-							
		undecanal			25.86			0.16				0.08			0.28					0.00				ND				-							
		benzene acetaldehyde			26.42			0.08				0.02			2.37					1.06				0.35				0.07							
		dodecanal			27.98			0.37				0.11			ND					-				ND				-							
		2-ethylbenzaldehyde			28.33			ND				-			0.09					0.02				ND				-							
		**Total**						**7.41**							**18.79**									**24.04**											
		*Esters*																																	
		ethyl acetate			3.04			ND				-			ND					-				0.13				0.04							
		3-hexen-1-ol-acetate			18.99			ND				-			ND					-				0.09				0.02							
		dodecanoic acid. methyl ester			29.70			ND				-			ND					-				ND				-							
		geranyl acetate			30.64			0.08				0.02			0.05					0.01				ND				-							
		**Total**						**0.08**							**0.05**									**0.21**											
		*Ketones*																																	
		2-heptanone			13.51			1.75				0.56			1.90					0.24				3.45				1.83							
		2-nonen-4-one			15.90			ND				-			ND					-				0.12				0.00							
		2-methyl-6-heptanone			16.21			0.44				0.33			0.31					0.03				0.42				0.02							
		3-octanone			16.77			ND				-			ND					-				0.11				0.00							
		acetoin			17.60			0.19				0.01			7.12^5^					0.13				6.81				1.87							
		2-octanone			17.87			ND				-			ND					-				1.13				1.18							
		pantoic lactone			24.39			0.95				0.03			0.20					0.10				0.07				0.02							
		**Total**						**3.33**							**9.52**									**12.1**											
		*Phenols*																																	
		anisole			19.5			ND				-			0.31					0.15				0.17				0.01							
		4-methyl anisole			22.01			3.53				1.01			0.15					0.01				2.40				0.18							
		phenol			31.58			0.15				0.02			ND					-				ND				-							
		4-methylphenol			33.27			0.23				0.01			13.25^1^					1.30				11.28^2^				1.23							
		**Total**						**3.91**							**13.7**									**13.84**											
		*Alcohols*																																	
		ethanol			3.82			ND				-			3.11					0.61				3.28				0.56							
		2-methyl-3-buten-2-ol			7.38			1.20				0.09			ND					-				ND				-							
		isobutanol			10.39			ND				-			0.64					0.31				0.12				0.00							
		1-butanol			12.71			ND				-			0.05					0.00				0.09				0.03							
		1-penten-3-ol			13.00			ND				-			ND					-				6.37				0.11							
		isoamylalcohol			15.47			0.10				0.00			13.03^2^					1.22				0.88				0.89							
		1-pentanol			16.98			ND				-			2.92					0.38				3.58				1.24							
		eptanol			19.20			0.52				0.12			0.27					0.02				0.47				0.18							
		3-hexen-1-ol			20.81			ND				-			0.14					0.02				8.52^3^				1.56							
		2-nonanol			20.87			2.60				0.67			0.46					0.10				ND				-							
		1-octen-3-ol			22.55			ND				-			1.39					0.46				0.32				0.19							
		1-octanol			24.93			0.08				0.05			ND					-				ND				-							
		1-nonanol			27.07			ND				-			1.18					0.09				ND				-							
		3-decen-1-ol			29.61			ND				-			ND					-				0.11				0.06							
		benzyl alcohol			30.91			ND				-			0.32					0.01				ND				-							
		**Total**						**4.5**							**23.5**									**23.74**											
		*Furans*																																	
		2-methylfuran			3.15			0.62				0.07			ND					-				ND				-							
		4-ethylfuran			4.21			ND				-			ND					-				5.22				1.43							
		**Total**						**0.62**							**ND**									**5.22**											
		*Terpenes*																																	
		α-pinene			6.03			47.95^1^				2.30			6.47^8^					0.04				6.23^5^				0.73							
		thujene			6.41			3.05				0.97			0.18					0.00				0.20				0.00							
		β-pinene			9.63			1.42				0.64			ND					-				0.37				0.05							
		sabinene			10.55			0.65				0.07			ND					-				0.20				0.18							
		β-myrcene			13.33			2.07				0.09			ND					-				0.58				0.57							
		terpene			14.39			2.83				0.25			ND					-				1.02				0.09							
		d-limonene			14.69			0.99				0.13			ND					-				2.91				1.27							
		γ-terpinene			16.40			0.46				0.00			ND					-				0.23				0.00							
		copaene			23.38			ND				-			1.93					1.56				ND				-							
		isolongifolene			23.89			ND				-			0.46					0.08				0.22				0.03							
		4-thujanol			24.58			0.51				0.08			0.27					0.00				0.12				0.03							
		linalool			24.71			ND				-			ND					-				0.30				0.01							
		quinhydrone			24.98			0.20				0.00			6.55^7^					0.04				0.17				0.08							
		caryophillene			25.61			0.07				0.02			0.18					0.07				0.08				0.27							
		4-terpineol			25.79			0.29				0.01			0.87					0.00				0.12				0.02							
		β-farnesene			27.21			ND				-			ND					-				ND				-							
		verbenone			27.64			0.24				0.03			ND					-				0.13				0.02							
		naphtalene			28.90			ND				-			ND					-				0.22				0.04							
		sesquiphellandrene			29.11			ND				-			ND					-				0.08				0.02							
		estragol			30.05			0.14				0.00			ND					-				ND				-							
		**Total**						**67.57**							**17.19**									**13.33**											
		*Free fatty acids*																																	
		acetic acid			22.27			0.24				0.07			0.07					0.03				ND				-							
		formic acid			23.46			ND				-			0.13					0.00				ND				-							
		propionic acid			24.27			ND				-			ND					-				ND				-							
		pivalic acid			25.21			0.04				0.00			ND					-				0.22				0.27							
		hexanoic acid			30.34			0.36				0.11			0.09					0.02				0.11				0.01							
		heptanoic acid			32.02			ND				-			0.14					0.05				1.34				0.18							
		octanoic acid			33.06			0.06				0.02			0.16					0.02				ND				-							
		nonanoic acid			34.13			0.19				0.05			0.31					0.01				0.14				0.06							
		**Total**						**0.88**							**0.91**									**1.81**											
		*Miscellaneous*																																	
		acetonitrile			5.42			2.37				1.03			0.63					0.20				0.36				0.03							
		nicotinonitrile			28.46			0.35				0.01			0.75					0.35				0.3				0.15							
		dimethyl sulfone			31.24			0.37				0.02			ND					-				ND				-							
		**Total**						**3.08**							**1.37**									**0.65**											

^a^ Retention time; ^b^ Normalized amount of volatile compounds (percentage) (peak of volatile compound/total peak area of all volatile compounds) of *O. sphegodes* subsp. *sphegodes*, *O. bertolonii* subsp. *benacensis* and *N. tridentata* flowering orchid samples (*n* = 3); ^c^ Standard deviation (±); ND: not detected; The most representative compounds for all orchids species were labeled with a number.

## 3. Experimental

### 3.1. Orchid Species Studied

*Ophrys sphegodes* Mill. subsp. *sphegodes,* commonly known as the Early Spider Orchid, is a very rare species that grows on alkaline and dry soils of Mediterranean coasts and mountain areas up to 1,200 m a.s.l. The plant is 25–50 cm tall and its inflorescence is formed by 4–6 flowers, which are characterized by yellow-green sepals and a velvety brown labellum with a distinctive H marking, so that the flowers very much resemble an arthropod and especially a spider. 

*Ophrys bertolonii subsp. benacensis* (Reisigl) P. Delforge is a rare sub-endemic species that grows on basic and poor grasslands between 80 to 750 m a.s.l., in northern Italy. This orchid is 20–30 cm tall and, like *O. sphegodes* subsp. *Sphegodes,* its inflorescence is formed by 4–6 flowers. The flowers have white or lilac sepals, green veined, pinkish purple petals and the labellum is brown marked with bluish or reddish spots. 

*Neotinia tridentata* (Scop.) R.M. Bateman, Pridgeon & M.W. Case is an Euro-Mediterranean species that grows in full sun on calcareous soils from sea level up to 1800 m a.s.l. The plant is 15–40 cm tall with a short, compact, ovoid inflorescence constituted by small, acuminate flowers. Sepals and petals are entirely lilac or pinkish purple veined, the labellum is trilobed, white to pale violet, marked with purple spots [28]. 

### 3.2. Orchid Population Sites Studied

**Figure 3:**
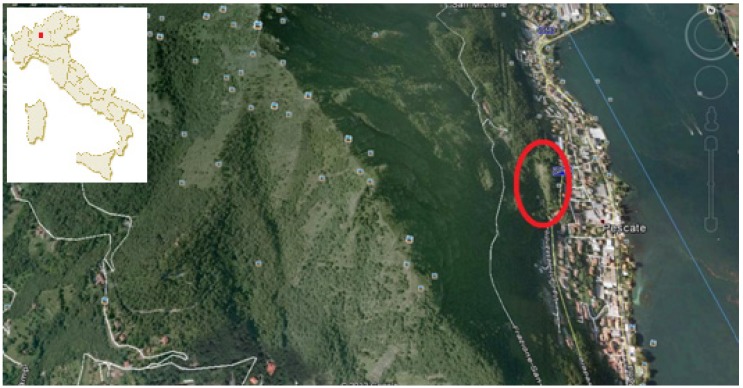
Pescate, Lecco, North of Italy and detailed view of the site of orchid sampling, Prato Olivino (Map from Google earth).

*O. sphegodes* subsp. *sphegodes*, *O. benacensis* and *N. tridentata* populations were identified [28] and sampled at flowering stage at the beginning of May 2013 in the area surrounding Prato Olivino, near Pescate, Lecco, Italy, (45°49’27.20” N; 9°23’53.54” E) located at an altitude of 280 m a.s.l. (Figure 3). According to the worldwide bioclimatic classification [29] the area belongs to the temperate oceanic bioclimate and to the low humid upper mesotemperate phytoclimatic belt. The habitat of orchids considered in this study is a semi-dry calcareous grassland (occasionally mown), belonging to the *Festuco-Brometea* Br.-Bl. & Tüxen ex Br.-Bl. 1949 class, *Brometalia erecti* Br.-Bl. 1936 order.

Prato Olivino is a natural area near the Monte Barro natural park, which can been considered, in accordance with the Directive 92/42/CEE, as an important orchid site, due to the presence of rare and endemic species [30].

### 3.3. Headspace Solid Phase Microextraction (HS-SPME) of Volatile Compound Sampling from Living Orchid Plants (in Vivo)

At the beginning of May 2013, *O. sphegodes* subsp. *sphegodes*, *O. benacensis* and *N.* *tridentata* plants were sampled in triplicate, *in vivo* and *in situ* in order to evaluate the volatile organic compound (VOC) emitted by living plants. Each plant was enclosed in an customised aerated glass cage manufactured by COLAVER s.r.l. (Vimodrone, MI, Italy), into which a manual SPME holder was inserted to extract the headspace. Volatile compounds were collected using a 50/30 µm divinylbenzene/Carboxen™/polydimethylsiloxane (DVB/CAR/PDMS) StableFlex™ fiber (Supelco, Bellefonte, PA, USA). The fibre was exposed to the plant headspace for 4 h. 

### 3.4. Headspace Solid Phase Microextraction (HS-SPME) of Volatile Compound Sampling from Flowering Orchid Plants

The HS-SPME extraction conditions were optimized in our previous study on the characterization of *Achillea collina* VOCs (selection of SPME fiber, sample amount, and extraction time, repeatability and precision of method) [31]. In this study flowering plant samples of all orchid species were picked and inserted into a 20 mL glass vial fitted with a cap equipped with a silicone/polytetrafluoroethylene septum (Supelco) in order to make the results comparable. Samples were prepared in triplicate. At the end of the sample equilibration period (1 h) a conditioned (1.5 h at 280 °C) 50/30 µm DVB/CAR/PDMS StableFlex™ fiber (Supelco) was exposed to the headspace of the sample for extraction (3 h) using a CombiPAL system injector autosampler (CTC Analytics, Zwingen, Switzerland). An extraction temperature of 30 °C was selected in order to prevent possible matrix alterations (oxidation of some compounds, particularly aldehydes) [32,33,34,35].

### 3.5. Gas Chromatography Mass Spectrometry Analysis of VOCs

HS-SPME analysis was performed using a Trace GC Ultra (Thermo-Fisher Scientific; Waltham, MA, USA) Gas Chromatograph coupled with a quadrupole Mass Spectrometer Trace DSQ (Thermo-Fisher Scientific; Waltham, MA, USA) and equipped with an Rtx-Wax column (30 m; 0.25 mm i.d.; 0.25 μm film thickness, Restek, PA, USA). The oven temperature program was: from 35 °C, hold 8 min, to 60 °C at 4 °C/min, then from 60 °C to 160 °C at 6 °C/min and finally from 160 °C to 200 °C at 20 °C /min. Carry over and peaks originating from the fiber were regularly assessed by running blank samples. After each analysis fibers were immediately thermally desorbed in the GC injector for 5 min at 250 °C to prevent contamination. The injections were performed in splitless mode (5 min). The carrier gas was helium at a constant flow of 1 mL·min^−1^. The transfer line to the mass spectrometer was maintained at 230 °C, and the ion source temperature was set at 250 °C. The mass spectra were obtained by using a mass selective detector with the electronic impact at 70 eV, a multiplier voltage of 1456 V, and by collecting the data at a rate of 1 scan·s^−1^ over the *m/z* range of 30–350. Compounds were identified by comparing the retention times of the chromatographic peaks with those of authentic compounds analyzed under the same conditions when available. The identification of MS fragmentation patterns was performed either by comparison with those of pure compounds or using the National Institute of Standards and Technology (NIST) MS spectral database. Volatile compound measurements from each headspace of orchid extracts were carried out by peak area normalization (expressed in percentage). All analyses were done in triplicate.

## 4. Conclusions

This study represented the first investigation regarding the VOC profile of different Italian populations of orchids using different pollinator attraction strategies, sampled *in vivo* and *in situ*. The results showed distinctive differences in volatile metabolite composition between orchids of the *Ophrys* and *Neotinea* genus. Moreover, a strong impact of the sampling methods on the volatile profiles, particularly regarding the different proportion of terpenes between picked flowering orchids and plants sampled *in vivo* and *in situ*, was observed. SPME could represent a good technique to analyze volatile compounds emitted by *in vivo* plants, sampled *in situ*, in a non-disruptive way, with potentially great advantages for phytochemical and ecophysiological studies, particularly regarding rare and/or protected plants, such as orchids.
